# Unmet medical needs in intermittent Claudication with diabetes and coronary artery disease—A “real‐world” analysis on 21 197 PAD patients

**DOI:** 10.1002/clc.23186

**Published:** 2019-05-06

**Authors:** Philipp Stalling, Christiane Engelbertz, Florian Lüders, Matthias Meyborg, Katrin Gebauer, Johannes Waltenberger, Holger Reinecke, Eva Freisinger

**Affiliations:** ^1^ Department of Cardiology I, Coronary and Peripheral Vascular Disease, Heart Failure University Hospital Muenster Muenster Germany; ^2^ Division of Vascular Medicine, Department of Cardiology I – Coronary and Peripheral Vascular Disease, Heart Failure Medicine University Hospital Muenster Muenster Germany; ^3^ AGZM Ambulantes Gefäßzentrum Münster Muenster Germany

**Keywords:** cardiovascular disease, clinical epidemiology, diabetes, health services research, patient centered outcomes research, prevention

## Abstract

**Background:**

Peripheral artery disease (PAD) is frequently co‐prevalent with coronary artery disease (CAD) and diabetes (DM). The study aims to define the burden of CAD and/ or DM in PAD patients at moderate stages and further to evaluate its impact on therapy and outcome.

**Methods:**

Study is based on health insurance claims data of the BARMER reflecting an unselected “real‐world” scenario. Retrospective analyses were based on 21 197 patients hospitalized for PAD Rutherford 1‐3 between 1 January 2009 to 31 December 2011, including a 4‐year follow‐up (median 775 days).

**Results:**

In PAD patients, CAD is prevalent in 25.3% (n = 5355), DM in 23.5% (n = 4976), and both CAD and DM in 8.2% (n = 1741). Overall, in‐hospital mortality was 0.4%, being increased if CAD was present (CAD alone: OR 1.849; 95%‐CI 1.066‐3.208; DM alone: OR 1.028; 95%‐CI 0.520‐2.033; CAD and DM: OR 3.115; 95%‐CI 1.720‐5.641). Both, CAD and DM increased long‐term mortality (CAD alone: HR 1.234; 95%‐CI 1.106‐1.376; DM alone: HR 1.260; 95%‐CI 1.125‐1.412; CAD and DM: HR 1.76; 95%‐CI 1.552‐1.995). DM further increased long‐term amputation risk (DM alone: HR 2.238; 95%‐CI 1.849‐2.710; DM and CAD: HR 2.199; 95%‐CI 1.732‐2.792), whereas CAD (alone) did not.

**Conclusions:**

In a greater perspective, the data identify also mild to modest stage PAD patients at particular risk for adverse outcomes in presence of CAD and/or DM. CAD and DM both are related with a highly increased risk of long‐term mortality even in intermittent claudication, and DM independently increased amputation risk.

## 1 INTRODUCTION

The prevalence of peripheral artery disease (PAD) increased dramatically by 13% in high‐income countries in only a decade, and is predicted to further rise worldwide with progressive aging of the population.^1^ A vast majority of PAD patients is at subclinical stages of the disease,^2^ yet at significant risk of cardiovascular events.^3^ Particularly the combination of diabetes mellitus (DM) and coronary artery disease (CAD) promotes development and progression of PAD.^2^, ^4^, ^5^, ^6^ Both, CAD and DM are linked in a complex manner with PAD outcome.^5^, ^6^, ^7^, ^8^, ^9^

Particularly lower limb calcification as mainly prevalent in DM patients is associated with increased cardiac mortality and morbidity.^5^, ^6^, ^10^ However, insulin‐dependent DM has shown to be an independent risk factor of adverse long‐term outcome also in ilio‐femoral PAD (all‐cause death at 5 years increased by 70%).^11^ The global prevalence of diabetic foot is at average 6.4%,^12^ corresponding to an incidence of 2% per year in a community‐based cohort of DM patients.^13^ As a consequence, the development of a diabetic foot syndrome dramatically worsens the prognosis in terms of limb salvage and death at all PAD stages.^8^

The predominant genesis of death in patients with mild to moderate PAD is of cardiovascular cause (approx. 40%).^12^ In addition, DM promotes the development of CAD as subsequent arteriosclerotic manifestation^6^, ^14^ leading to a notable co‐prevalence of DM in polyvascular disease in approx. 35%.^15^ CAD is reported to be concomitant in approx. About 40% to 80% of PAD patients with intermittent claudication.^16^, ^17^ In this context it is of particular importance that PAD on its part is associated with increased severity of CAD as indicated by higher rate of multivessel and left main CAD.^18^, ^19^ Heart failure is therefore more common in PAD and reduced left ventricular ejection fraction increased risk of major cardiovascular events in patients with intermittent claudication.^20^

The detrimental prognosis even in low‐stage PAD with progression from intermittent claudication to critical limb ischemia in 21% of cases, and need of amputation in 4% to 27% of claudicants within 6 years,^21^ sets focus on risk factor management as a matter of particular importance.

We therefore aimed to investigate the actual impact of concomitant DM and/or CAD on management of care and outcome in low‐stage PAD patients (Rutherford grade 1‐3) in a real‐life scenario on a large scale.

## 2 METHODS

The retrospective analysis is based on routine data provided by the BARMER GEK health insurance and consists of 21 197 hospitalized patients with subclinical to moderate peripheral artery disease (PAD). Representing about 10% of the entire German population the BARMER GEK covers approximately 8.7 million insured patients. All German hospitals are obliged to transfer data on diagnoses, co‐morbidities and complications to the health insurances in form of detailed and obligatory coding guidelines: German Modification of the International Statistical Classification of Diseases and Related Health Problems 10th Revision (ICD‐10‐GM). For hospital reimbursement, all diagnostic and therapeutic procedures have to be coded according to the German procedure classification (“Operationen und Prozedurenschlüssel”, OPS). The German Diagnosis related Groups (G‐DRG) system is used for hospital reimbursement in Germany since 2004. Annual adaptations of the ICD‐10‐GM were accounted for in the study. After patients' discharge, one code for the main diagnosis must be selected reflecting the underlying cause for admission. For additive coding of secondary diagnoses, a vast number of supplementary codes can be chosen representing co‐morbidities and complications which were present or occurred during in‐hospital stay. Reimbursement is calculated on the basis of patients' extent of diagnoses and the procedures performed. In general, factors enhancing the reimbursement (CKD, DM, CHF, symptomatic PAD, malignancies, acute renal failure, in‐hospital infections, and sepsis) are unlikely to be omitted, since only complete coding is the essential condition for correct reimbursement and therefore of economic importance for the hospitals. About 20% of the codes are controlled—and corrected if required—by specialized physicians (“Medizinischer Dienst der Krankenversicherung”) independently from health insurances and hospitals.

The anonymized data of the BARMER GEK health insurance are accessed as previously described^9^:

We analyzed a total of 21 197 in‐hospital patients with primary or secondary diagnosis of PAD at Rutherford grades 1‐3 (corresponding to intermittent claudication; ICD 10 GM 2011: I70.20 and I70.21). The analysis includes all patients with an index‐hospitalization between 1 January 2009 and 31 December 2011, including a follow‐up period of at least 24 months after discharge until 31 December 2012 (median FU‐period: 775 days; 25th‐75th percentiles 469‐1120 days).

Within these Rutherford grades (ICD 10 I70.20 and I70.21), subgroups with co‐diagnoses of diabetes mellitus (DM; IDC‐10 E10*, E11*) and coronary artery disease (CAD; ICD‐10 I25* and/or previous coronary artery bypass grafting OPS 5.360‐3) have been identified. Further, co‐diagnoses of hypertension, obesity, dyslipidemia, smoking, chronic kidney disease, chronic heart failure, and malignancies have been ascertained according to ICD‐10 coding. Additionally, complications specifically acute renal failure, acute myocardial infarction, ischemic stroke, infection, and sepsis as well as in‐hospital mortality have been assessed accordingly. A detailed specification of definitions and ICD‐10 codes is given in the supplements (Supporting Information Table S1). Procedures during index‐hospitalization have been defined by use of the OPS and specified as (diagnostic) angiography, any revascularization (endovascular and/or surgical), endovascular revascularization (EVR), surgical revascularization, thrombendarterectomy (TEA), peripheral bypass grafting, and amputation (major, minor, recurrent). A detailed specification on the diagnostic and procedural codes is presented in the supplements (Table S2).

### 2.1 Statistics

Patients with mild to moderate PAD as defined above were divided into four subgroups according to co‐diagnosis of DM and/or CAD: PAD no DM no CAD co‐diagnosis; PAD with DM but no CAD; PAD with CAD but no DM; PAD with both DM and CAD.

Within these subgroups, specific (co‐)diagnoses and procedures are stated as absolute numbers (n) and percentages (%) of the respective sub‐cohort. Categorical variables were statistically compared by use of the chi‐square test. Continuous variables were shown as mean ± SD and were analyzed by the anova‐*F*‐test. *P* values of <0.05 were considered as statistically significant. The influence of baseline variables on in‐hospital outcome parameters were tested by binary logistic regression models and were displayed as OR with 95%‐CI. Long‐term outcome parameters were tested by multivariable Cox‐regression analysis (covariates: age, gender, hypertension, obesity, dyslipidemia, smoking, CKD, CHF, and malignancies) and its results were displayed as HR with 95%‐CI, and cumulative event curves.

## 3 RESULTS

A total of 21 197 patients with mild to moderate PAD (Rutherford classification: Ruth 1‐3) were included in the analysis. Of these, 5355 (25.3%) suffered from concomitant CAD, 4976 (23.5%) from concomitant DM, and 1741 (8.2%) from both CAD and DM. Baseline characteristics of PAD patients with regard to CAD and DM are presented in Table 1. The proportion of male patients increased from 53.6% in PAD without DM or CAD, to 61.6% with DM alone, to 67.1% with CAD alone, to 71.8% in PAD patients with DM and CAD. Co‐morbidity with DM and CAD was further associated with increased age (71.8 years vs 53.6 years in PAD without DM or CAD; *P* < 0.001) and high prevalence of common cardiovascular risk factors (eg, hypertension 83.1%; obesity 15.9%; dyslipidemia 54.4%; chronic heart failure 13.7%), but not smoking (7.1% vs 17.4% in PAD without DM or CAD; *P* < 0.001). Previous ischemic stroke was particularly present in PAD patients with concomitant DM (2.2% PAD with DM alone; 2.9% PAD with DM and CAD), whereas chronic kidney disease (CKD) was equally increased by DM (18.1%) or CAD (19.8%) to 31.2% in PAD with DM and CAD.

**Table 1:** Baseline characteristics and co‐morbidities with regard to CAD and DM status

	No CAD no DM	No CAD DM	CAD no DM	CAD DM	All	*P*
Patients, n (% of all)	12.607 (59.5)	3235 (15.3)	3614 (17.0)	1741 (8.2)	21 197 (100.0)	
Age, mean ± SD	67.5 ± 10.8	69.2 ± 9.9	70.3 ± 9.8	71.0 ± 8.7	68.5 ± 10.4	**<0.001**
Men, n (%)	6762 (53.6)	1991 (61.6)	2423 (67.1)	1249 (71.8)	12 425 (58.6)	**<0.001**
CAD^a^, n (%)	0 (0.0)	0 (0.0)	3614 (100.0)	1741 (100.0)	5355 (25.3)	**<0.001**
Previous MI, n (%)	0 (0.0)	0 (0.0)	434 (12.0)	188 (10.8)	622 (11.6)	**<0.001**
Previous CABG, n (%)	0 (0.0)	0 (0.0)	268 (7.4)	114 (6.5)	382 (7.2)	**<0.001**
**Cardiological risk factors**						
Hypertension, n (%)	7746 (61.4)	2529 (78.2)	2945 (81.5)	1447 (83.1)	14 667 (69.2)	**<0.001**
Obesity, n (%)	626 (5.0)	397 (12.3)	258 (7.1)	276 (15.9)	1557 (7.3)	**<0.001**
Dyslipidemia, n (%)	3860 (30.6)	1234 (38.1)	1900 (52.6)	947 (54.4)	7941 (37.5)	**<0.001**
Diabetes mellitus, n (%)	0 (0.0)	3235 (100.0)	0 (0.0)	1741 (100.0)	4976 (23.5)	**<0.001**
Smoking, n (%)	2190 (17.4)	404 (12.5)	410 (11.3)	123 (7.1)	3127 (14.8)	**<0.001**
CHF, n (%)	284 (2.3)	160 (4.9)	339 (9.4)	238 (13.7)	1021 (4.8)	**<0.001**
**Cardiovascular co‐morbidities**						
Previous ischemic stroke, n (%)	193 (1.5)	72 (2.2)	67 (1.9)	50 (2.9)	382 (1.8)	**<0.001**
CKD, n (%)	1179 (9.4)	585 (18.1)	716 (19.8)	543 (31.2)	3023 (14.3)	**<0.001**
Malignancies, n (%)	137 (1.1)	37 (1.1)	67 (1.9)	20 (1.1)	261 (1.2)	**0.003**
**Vascular status**						
Previous EVR, n (%)	820 (6.5)	244 (7.5)	357 (9.9)	188 (10.8)	1609 (7.6)	**<0.001**
Previous vascular surgery, n (%)	133 (1.1)	40 (1.2)	54 (1.5)	18 (1.0)	245 (1.2)	0.161
Previous amputation, n (%)	35 (0.3)	32 (1.0)	9 (0.2)	14 (0.8)	90 (0.4)	**<0.001**

Abbreviations: CAD, coronary artery disease; CHF, chronic heart failure; CKD, chronic kidney disease; DM, diabetes mellitus; EVR, endovascular revascularization; MI, myocardial infarction.

a CAD as the main diagnosis according to ICD code I25 and/or previous myocardial infarction and/or coronary artery bypass grafting.

Overall, 7.6% of all PAD patients at Ruth 1‐3 had undergone previous endovascular revascularization procedures (EVR). Particularly CAD was associated with increased previous EVR (PAD Ruth 1‐3:10.8% in CAD and DM vs 9.9% in CAD alone vs 7.5% in DM alone vs 6.5% in PAD without DM or CAD; *P* < 0.001). In contrast, previous vascular surgical procedures (overall 1.2%) did not differ significantly in these subgroups (Table 1). Concomitant DM was associated with previous limb amputation (1.0% DM alone, 0.8% DM and CAD) compared to non‐diabetic PAD patients (0.2% in CAD alone; 0.3% without DM or CAD; *P* < 0.001).

### 3.1 In‐hospital treatment and complications

During index‐hospitalization, there were significant differences regarding treatment, complications, and outcome in dependence on CAD and DM co‐diagnoses (Table 2).

**Table 2:** Treatment, complications, and outcomes during index‐hospitalization

	No CAD no DM	No CAD DM	CAD no DM	CAD DM	All	*P*
Patients, n (% of all)	12.607 (59.5)	3235 (15.3)	3614 (17.0)	1741 (8.2)	21 197 (100.0)	
***Treatment***						
Angiography, n (%)	7420 (58.9)	1794 (55.5)	2173 (60.1)	952 (54.7)	12 339 (58.2)	**<0.001**
Any revascularization, n (%)	9901 (78.5)	2252 (69.6)	2688 (74.4)	1122 (64.4)	15 963 (75.3)	**<0.001**
EVR, n (%)	7244 (57.5)	1638 (50.6)	1909 (52.8)	811 (46.6)	11 602 (54.7)	**<0.001**
Vascular surgery, n (%)	3100 (24.6)	702 (21.7)	889 (24.6)	377 (21.7)	5068 (23.9)	**0.001**
TEA, n (%)	1594 (51.4)	381 (54.3)	534 (60.1)	227 (60.2)	2736 (54.0)	**0.001**
Bypass, n (%)	1316 (42.5)	277 (39.5)	333 (37.5)	142 (37.7)	2068 (40.8)	**<0.001**
***Complications***						
Acute renal failure, n (%)	33 (0.3)	12 (0.4)	18 (0.5)	13 (0.7)	76 (0.4)	**0.006**
MI, n (%)	7 (0.1)	4 (0.1)	35 (1.0)	22 (1.3)	68 (0.3)	**<0.001**
Ischemic stroke, n (%)	15 (0.1)	7 (0.2)	7 (0.2)	4 (0.2)	33 (0.2)	0.422
Infections, n (%)	185 (1.5)	147 (4.5)	69 (1.9)	90 (5.2)	491 (2.3)	**<0.001**
Sepsis, n (%)	37 (0.3)	20 (0.6)	15 (0.4)	16 (0.9)	88 (0.4)	**<0.001**
Amputations, n (%)	29 (0.2)	42 (1.3)	9 (0.2)	23 (1.3)	103 (0.5)	**<0.001**
***Outcome***						
In‐hospital mortality, n (%)	41 (0.3)	11 (0.3)	22 (0.6)	19 (1.1)	93 (0.4)	**<0.001**
In‐hospital stay, mean (95% CI), days	5.3 (5.2‐5.4)	6.5 (6.2‐6.8)	5.9 (5.7‐6.2)	7.5 (7.1‐8.0)	5.8 (5.7‐5.9)	**<0.001**
In‐hospital stay, median, days	3.0	4.0	3.0	5.0	3.0	
Reimbursement costs, mean (95%CI), €	3581(3531‐3630)	3673(3560‐3786)	3742(3634‐3851)	4063(3844‐4282)	3662(3619‐3705)	**<0.001**
Reimbursement costs, median, €	2700	2711	2724	2763	2710	

Abbreviations: CAD, coronary artery disease; CI, confidence interval; DM, diabetes mellitus; EVR, endovascular revascularization; MI, myocardial infarction; TEA thrombendatherectomy.

Diagnostic angiography was performed in 58.2% of all PAD patients, and was significantly decreased in subgroups with DM (55.5% in DM alone and 54.7% in DM and CAD compared to 60.1% in CAD alone; *P* < 0.001). Of all PAD patients, 75.3% received a revascularization procedure (54.7% EVR, and 23.9% surgical). EVR was performed at lower frequency in subgroups with CAD alone (52.8%), DM alone (50.6%) and at most by the combination of CAD and DM (46.6%; *P* < 0.001; Table 2). Particularly DM patients received significantly less surgical revascularization than those without DM, however bypass surgery was performed less often in patients with concomitant CAD.

The rate of severe in‐hospital complications was at low level with infection as the leading diagnosis (n = 491 patients; 2.3%; see Table 2). Local infection, sepsis, and amputation were more common in patients with DM than in non‐diabetic PAD patients (*P* < 0.001). Whereas concomitant DM increased the length‐of‐stay (4 days vs 3 days without DM), CAD severely increased costs of the index‐hospitalization (3742 EUR CAD alone vs 3673 EUR in DM alone vs 3581 EUR in no DM no CAD; *P* < 0.001; Table 2). During index‐hospitalization, a total of 103 patients (0.5%) were amputated. DM increased the risk of amputation 1.7‐fold (n = 42, 1.3% vs n = 29, 0.2% in non‐DM PAD patients). The prevalence of CAD had no major impact on the amputation‐rate. The overall in‐hospital mortality was 0.4% (n = 93) in the entire PAD cohort (Table 2). Concomitant DM alone was not associated with increased in‐hospital mortality (0.3% DM alone vs 0.3% in no DM no CAD), however concomitant CAD increased in‐hospital mortality to 0.6% in CAD alone and up to 1.1% in PAD patients with both, CAD and DM (*P* < 0.001). Multivariate binary regression analysis showed CAD alone to independently increase the risk of in‐hospital mortality having an odds ratio (OR) of 1.849 (95%‐CI 1.066‐3.208; *P* = 0.029; Table 3), and to be further increased by additional DM co‐diagnosis (OR 3.115; 95%‐CI 1.720‐5.641; *P* < 0.001).

**Table 3:** Univariable and multivariable binary logistic regression analysis of in‐hospital mortality

	Unadjusted OR (95% CI)	*P*	Adjusted^a^ OR (95% CI)	*P*
No CAD, no DM	1	**<0.001**	1	**0.001**
No CAD, DM	1.046 (0.537‐2.037)	0.895	1.028 (0.520–2.033)	0.936
CAD, no DM	1.877 (1.117‐3.155)	**0.017**	1.849 (1.066–3.208)	**0.029**
CAD, DM	3.382 (1.958‐5.840)	**<0.001**	3.115 (1.720–5.641)	**<0.001**

Abbreviations: CAD, indicates coronary artery disease; CHF, chronic heart failure; CKD, chronic kidney disease; DM, diabetes mellitus.

a Adjusted for age, sex, hypertension, obesity, dyslipidemia, smoking, CKD, CHF malignancies.

### 3.2 Long‐term outcome

**Figure 1:**
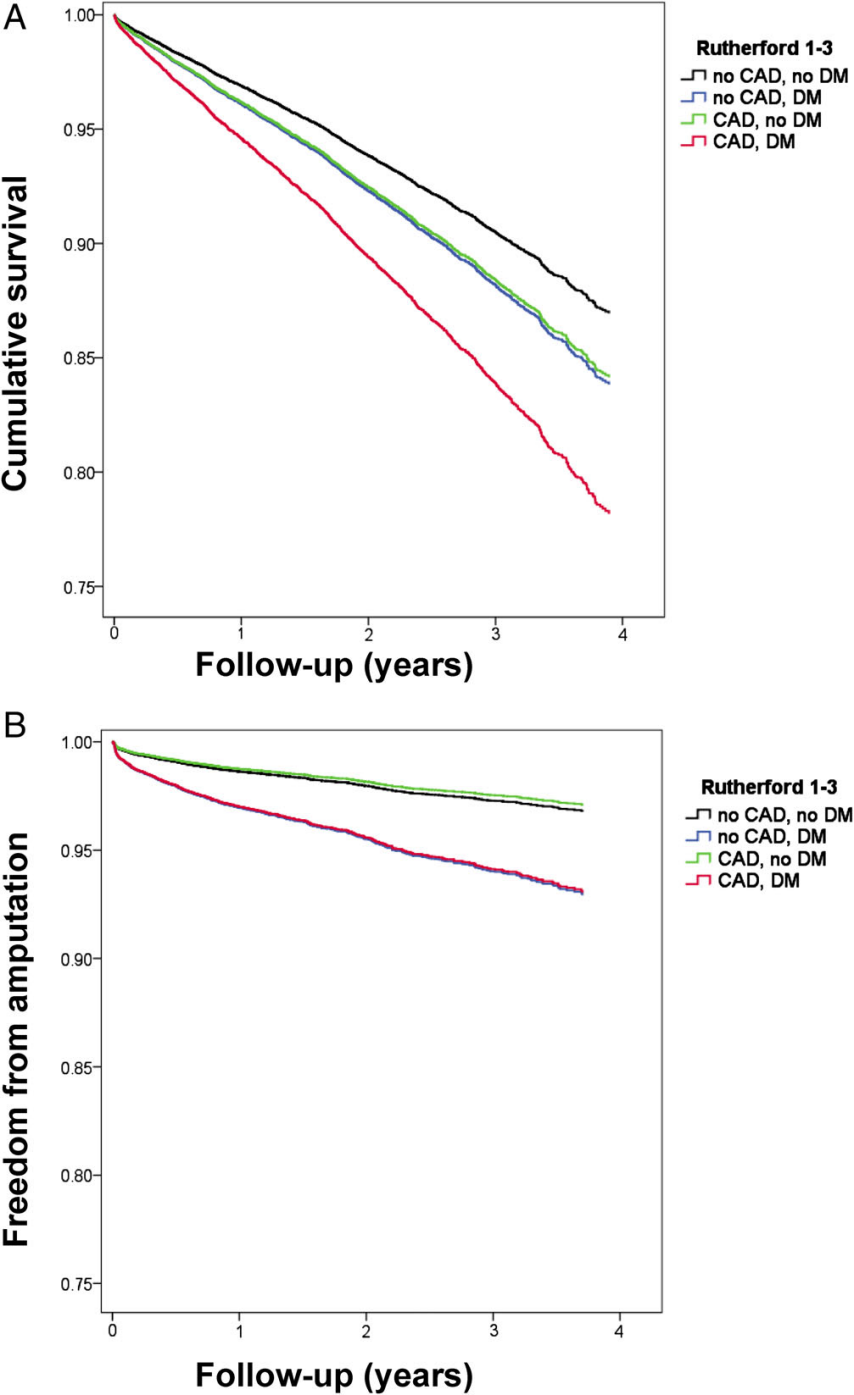
Long‐term overall survival and freedom from amputation in PAD patients depending on concomitant CAD or DM. Cox‐regression analysis adjusted for co‐morbidities and baseline parameters in PAD subgroups without concomitant DM/CAD (black), with DM only (blue), with CAD only (green), and both DM and CAD (red) is shown for outcome parameters amputation (panel A) and mortality (panel B). Panel A, Overall survival was about equally deteriorated by each, DM and CAD alone, and further worsened if DM and CAD combined. Panel B, Freedom from amputation was significantly reduced by concomitant DM irrespective of the presence of CAD. Amputation‐free survival was about equal in patients with neither CAD nor DM co‐diagnosis and in concomitant CAD alone. Concomitant DM alone decreased amputation‐free survival irrespective of additional diagnosis of CAD. CAD, coronary artery disease; DM, diabetes mellitus; PAD, peripheral artery disease

The long‐term mortality and amputation were assessed during the follow‐up period of up to 4 years (median 775 days; 25th‐75th percentiles 469‐1120 days). During follow‐up, amputations were performed in 664 patients (3.1%). PAD patients with DM had significantly higher amputation rates than patients without DM (no CAD, no DM: 2.3%; CAD, no DM: 2.4%; no CAD, DM: 5.6%; CAD and DM 6.2%; *P* < 0.001; Figure 1A). In the multivariate Cox regression, DM was an independent risk factor of amputation during follow‐up (hazard ratio HR 2.238; 95%‐CI 1.849‐2.710; *P* < 0.001; Table 4) whereas CAD (alone) was not (HR 0.907, *P* = 0.445).

**Table 4:** Multivariable Cox‐regression analysis of mortality and amputation during follow‐up

	Amputation		Mortality	
	Adjusted^a^ HR (95% CI)	*P* value	Adjusted^a^ HR (95% CI)	*P* value
no CAD, no DM	1	**<0.001**	1	**<0.001**
no CAD, DM	2.238 (1.849–2.710)	**<0.001**	1.260 (1.125–1.412)	**<0.001**
CAD, no DM	0.907 (0.708‐1.164)	0.445	1.234 (1.106–1.376)	**<0.001**
CAD, DM	2.199 (1.732–2.792)	**<0.001**	1.760 (1.552–1.995)	**<0.001**

Abbreviations: CAD, indicates coronary artery disease; CHF, Chronic heart failure; CKD, chronic kidney disease; DM, diabetes mellitus.

a Adjusted for age, sex, hypertension, obesity, dyslipidemia, smoking, CKD, CHF malignancies.

A total of 2447 (11.5%) PAD patients died during follow‐up. Mortality rates highly increased with co‐diagnosis of CAD and/or DM (no CAD, no DM: 9.2%; no CAD, DM: 13.0%; CAD, no DM: 13.9%; CAD and DM 20.9%; *P* < 0.001). Both, CAD (HR 1.234; 95%‐CI 1.106‐1.376; *P* < 0.001) and DM (HR 1.260; 95%‐CI 1.125‐1.412; *P* < 0.001) independently increased long‐term mortality with highest risk if combined (HR 1.76; 95%‐CI 1.552‐1.995; *P* < 0.001; Table 4 and Figure 1B).

## 4 DISCUSSION

This present study of an unselected cohort of 21 197 hospitalized patients at moderate stage of PAD highlights several aspects of the concomitant prevalence and impact of CAD and DM.

First, patients with PAD are at substantial risk of in‐hospital mortality and amputation even at low clinical PAD stages.

Second, concomitant DM was associated with reduced revascularization rates in claudicants and 2.3‐fold increased risk of amputation.

Third, long‐term mortality increased to 21% in PAD with DM and CAD compared to 9% in patients without during 4‐year follow‐up (HR 1.76; 95%‐CI 1.552‐1.995; *P* < 0.001).

Patients with intermittent claudication without concomitant DM or CAD are at average younger and generally at healthier condition with regard to classical cardiovascular risk factors. Despite that, the percentage of hypertension in two‐thirds and dyslipidemia in one‐third of patients, as well as the highest share of active smokers in this subgroup (17%) points at challenging conditions for secondary prevention.

Concomitant DM was present in 23.5%, CAD in 25.3%, and both CAD and DM in 8% of all hospitalized PAD patients at Rutherford 1‐3. These numbers correspond well with the data reported by the get ABI cohort study on low‐stage PAD in the ambulatory sector (85% asymptomatic PAD, concomitant DM 23.2%, concomitant CVD 27.7%).^2^ Despite commonly the ratio of female patients increases particularly in higher age groups,^22^, ^23^, ^24^ the combination of PAD claudicants with either DM or CAD leads to a selection of a predominantly male patient cohort. The distribution of cardiovascular risk factors correspond well with other data on cardiovascular disease (CVD) in general.^25^, ^26^

### 4.1 Impact of DM and CAD on amputation and death in low‐stage PAD patients

DM was associated with previous amputation in already 1% of claudicants (compared to 0.2% in PAD patients with CAD), reflecting the relatively high risk of foot ulceration even in preserved perfusion of the limb (annual incidence of foot ulceration in diabetics 2%).^12^, ^13^ The high impact of DM on limb loss in PAD becomes further evident in view of recent data on patients undergoing major amputation despite relatively high preoperative ABI‐values of 0.78.^27^ Of these, 67% had concomitant diabetes, and 32% had coronary artery disease. Comparably, our data show co‐morbidity with DM to increase the risk of in‐hospital amputation 6‐fold and during follow‐up 2‐fold compared to concomitant CAD only.

Although the actual need for revascularization in mild to modest stage PAD may be in principle questioned,^28^ DM patients might differ as a population at particular high risk. In terms of feasibility, recent population‐based data display a trend towards increased use of revascularization techniques followed by improved limb salvage in claudicants with infrainguinal limb revsacularization.^29^ However, previous EVR was conducted in only 7.5% of these moderate stage PAD patients with concomitant DM (compared to 9.9% in PAD and CAD) potentially due to delayed or atypical leg symptoms in DM patients.^30^ Also, during in‐hospital stay, DM was associated with lower rate of angiography and overall revascularization. Against the background of infections as the leading complication (up to 5.2% in PAD patients with CAD and DM), it should be noticed that the risk of infection has been reported 8fold increased for bypass surgery compared to EVR,^31^ indicating to favor a minimal invasive therapy particularly in DM patients.

In‐hospital mortality was generally low (up to 1.1%), however presence of CAD was independently associated with increased death during in‐hospital (OR 1.849; *P* = 0.029) and follow‐up period (HR 1.234; *P* < 0.001) in line with current knowledge.^28^, ^32^ Whereas a clear benefit from revascularization on survival rates is known for PAD patients with critical limb ischemia,^9^, ^28^ it remains to be proven for PAD with intermittent claudication.

Nevertheless, given the fate of claudicants with an average 4‐year‐mortality rate of 11.5% in the overall cohort (15%‐30% at 5‐years in the literature^33^;) and further increase up to 21% with concomitant DM and CAD, this is a clear call to action.

Not only that recognition of early stage PAD remains a matter of particular significance but also is consequent treatment of risk factors and timely prevention.^34^, ^35^ Finally, further research is imperative on how to effectively address the adverse impact of DM and CAD as major predictors of limb loss and mortality in PAD with intermittent claudication.

### 4.2 Strengths and limitations

Strengths of our study is its large size of 21 197 unselected PAD patients reflecting “real‐life scenario” including a 4‐year follow‐up period. Diagnoses and procedures are based on encoded data using the German DRG and OPS code. Despite generally high data integrity and validity, inaccuracy within the limits of the encoding systems may have occurred. PAD diagnosis in routine‐data may hypothetically also include asymptomatic PAD since these are not clearly to be separated from mild intermittent claudication by ICD‐10 GM 2011. However, encoding asymptomatic PAD did not lead to higher reimbursement category as potential inducement for over‐coding. Further, DM and CAD may be under‐diagnosed in early PAD stages. These under‐recognitions would result in an underestimation of the presented hazards.

Finally, information on underlying reasons for therapeutic decisions, the technical success of the procedures or concomitant medication is not included in the data set.

## 5 FUTURE DIRECTIONS

PAD patients are already at mild to moderate stage of disease at particular risk of death and amputation in the presence of CAD and/or DM. Future research is needed to clarify the benefits from early and consistent risk factor control in these patients. Particularly the role of endovascular therapy at early PAD stages with cardiovascular risk constellations should be addressed by future research to effectively prevent amputation.

## 6 CONCLUSION

Our study revealed a highly adverse impact of concomitant DM and CAD on the fate of PAD patients even at subclinical to moderate stages of disease. CAD was related with increased risk of short‐ and long‐term mortality, whereas DM highly increased amputation risk against the background of relatively low performance of revascularizations.

## CONFLICT OF INTERESTS

The authors PS, NM, CE, FL, KG, MM, and EF have no conflicts of interest. JW reports personal fees and non‐financial support from Biotronik, Boehringer Ingelheim, Daiichi‐Sankyo as well as personal fees from Bayer Vital, MSD, Berlin‐Chemie, and Vifor outside the submitted work. HR reports personal fees from NovoNordisk, Pluristem, Daichii, BMS, DiaPlan, MedUpdate, and Pfizer outside the submitted work.

## AUTHORS' CONTRIBUTIONS

PS and EF were responsible for data analysis and interpretation and wrote the manuscript. NM and HR advised on study design and provided careful review of the manuscript. CE was responsible for primary data analysis. FL, KG, MM and JW provided careful review of the manuscript and data interpretation. All authors read and approved the final manuscript.

## Supporting information


**Table S1.** ICD‐10 GM diagnostic codes
**Table S2.** OPS procedure codes
