# Supplementary material for: Unmet medical needs in intermittent Claudication with diabetes and coronary artery disease—A “real‐world” analysis on 21 197 PAD patients
Source: Clin Cardiol. 2019 May 6;42(6):629–36. doi: 10.1002/clc.23186 (PMC6553564; doi:10.1002/clc.23186)
Supplement: Supplementary file 1 — Table S1. ICD‐10 GM diagnostic codes Table S2. OPS procedure codes [file CLC-42-629-s001.pdf]

Stalling et al. **Unmet medical needs in Intermittent Claudication with Diabetes and Coronary Artery Disease - a ‚real-world' analysis on 21,197 PAD patients**

Table S1: ICD-10 GM diagnostic codes

ICD code	Description
Definition of peripheral artery disease (PAD) at Rutherford grades 0-3	
main diagnosis I70.20	Lower- limb PAD: asymptomatic or walking distance >200 m
main diagnosis I70.21	Lower limb: Exercise induced pain with walking distance < 200 m
co-diagnoses of I70.20 and I70.21 (Ruth 0-3) combined with qualifying diagnoses (*see below*):	
Codes for qualifying diagnoses in combination with co-diagnoses I70.20 and I70.21	
E10.50, E10.51	Type I diabetes with peripheral vascular complications
E10.74, E10.75	Type I diabetes with diabetic foot syndrome
E11.50, E11.51	Type II diabetes with peripheral vascular complications
E11.72, E11.73	Type II diabetes with multiple including vascular complications
E11.74, E11.75	Type II diabetes with diabetic foot syndrome
I70.25	Atherosclerosis of arteries of upper extremities
I73*	Other peripheral vascular diseases
I74*	Arterial embolism and thrombosis
L03.01, L03.02, L03.11	Cellulitis of finger and toe including acute lymphangitis
L98.4	Chronic ulcer of skin, not elsewhere classified
R02	Gangrene, not elsewhere classified
Codes for co-diagnoses and complications	
A30-49*	Other bacterial diseases including systemic inflammatory response syndrome
B95-B99*	Bacterial, viral and other infectious agents
C*	Neoplasms
E10*, E11*	Diabetes mellitus (Type I, II)
E66*	Obesity
E78*	Dyslipidemia
F17*	Nicotine abuse

I10-15*	Arterial hypertension
I21*	Acute myocardial infarction
I25*	Chronic ischaemic heart disease (CAD)
I50*	Chronic heart failure
I63*	Cerebral infarction incl. occlusion and stenosis of cerebral and precerebral arteries, resulting in cerebral infarction
I70.2*	Atherosclerosis of arteries of extremities
N17*	Acute renal failure
N18*	Chronic kidney disease

Diagnosis codes used for patients' identification and inclusion in the analysis are printed in the upper half of the table. Codes for analysis of secondary diagnoses, comorbidities and complications during in-hospital stay are shown in the lower part of the Table.
Of note, the German ICD-10 catalogue is in all cases identical with the WHO ICD-10 catalogue, but provides at some points more detailed subdiagnoses and descriptions for correct coding with the German DRG system. The * indicates that additional digits for specific descriptions of types and locations must be added. ICD-10 indicates International classification of Disease 10th version; PAD, peripheral arterial disease.

Table S2: OPS procedure codes

OPS Code	Description
Angiography	
3-605	Diagnostic angiography of iliac vessels
3-607	Diagnostic angiography of lower limb vessels
Endovascular revascularization (EVR)	
8-836*	Endovascular treatment (e.g. PTA, thrombolysis)
8-84*	Endovascular stenting
Vascular Surgery	
5-380*	Incision, embolectomy and thrombectomy
5-381*	Thrombendatherectomy (TEA)
5-383*	Resection and interponats of blood vessels
5-386*	Excision of aneurysms
5-388*	Suture of vascular vessels
5-393*	Peripheral bypass grafting
5-395*	Patch operations
Amputation	
5-864*	Major amputations, above the ankle
5-865*	Minor amputations, below the ankle
5-866*	Revision of an amputation

All procedures and interventions have to be coded with a distinct code for type and anatomic location of the treatment, which then is relevant for correct grouping and reimbursement in the German DRG system. The * indicates that additional digits for specific descriptions of types and anatomic locations can/must be added. OPS indicates “Operationen und Prozedurenschlüssel”.
